# Unintended pregnancy in the amazon basin of Ecuador: a multilevel analysis

**DOI:** 10.1186/1475-9276-9-14

**Published:** 2010-06-03

**Authors:** Isabel Goicolea, Miguel San Sebastian

**Affiliations:** 1 Umeå International School of Public Health, Department of Public Health and Clinical Medicine, Umeå University, SE-90185, Sweden; 2 Umeå Centre for Gender Studies, Umeå University, SE-90185, Sweden

## Abstract

**Background:**

It has been estimated that each year 80 million women in the world experience an unintended pregnancy. In Ecuador, recent research has revealed that 36.3% of total births are unintended; the research also details significant geographical, ethnic and socioeconomic variations. These studies focused on individual risk factors and were based on large national surveys where local samples, particularly from rural remote areas, were small. The purpose of this study was to investigate the influence of contextual and individual factors on unintended pregnancies in the Amazon Basin of Ecuador.

**Methods:**

Women aged 15-44 were selected through an ongoing community-based cross-sectional survey conducted in the Orellana province between May and December 2006. Data were fitted using multilevel logistic regression, adjusting for both individual-level and community-level factors as fixed effects and allowing for heterogeneity between communities.

**Results:**

The overall prevalence of unintended pregnancy was 62.7%. Two-thirds (73.7%) of indigenous women reported having had at least one unintended pregnancy. Being young, single, and indigenous were significant risk factors for unintended pregnancy, alongside having low access to education and having more than two children. No relationship was found between socioeconomic status and the use of contraceptives. All the variation between communities was explained by individual-level factors.

**Conclusions:**

This study showed the significance of individual factors in increasing the risk of unintended pregnancy, while the role of community factors was found to be negligible. In order for all women to be able to realize their right to reproductive autonomy, there needs to be a diverse range of solutions, with particular attention paid to cultural issues.

## Background

It has been estimated that each year 80 million women in the world experience unintended pregnancy. Unintended pregnancy (both unwanted and mistimed) increases the risk of abortion-related morbidity and mortality [1]; this is especially significant in countries where abortion remains illegal, which is the case in most Latin American countries [2]. Latin America and the Caribbean show the highest incidence rate of unsafe abortion worldwide with 32 unsafe abortions per 100 live births and an estimated 3,700,000 unsafe procedures carried out each year [3]. Unintended pregnancy is also associated with negative impacts on antenatal care, breastfeeding, child nutrition, and infant mortality. The effects on the mother's health have not been researched in any depth, but the existing studies show an increased risk of depression and anxiety [4,5]. In low-income countries, low use of contraception continues to be the main factor influencing the prevalence of unintended pregnancy [6]. Low contraception use has been linked to poor access to reproductive health services, gender norms, and sexual abuse or coercion [1,7].

Unintended pregnancy is also a rights issue. One fundamental reproductive right is the right to be in control of one's own fertility. The exercise of this right depends not only on ensuring access to information and actual contraception, but also on the individual freedom to make decisions regarding sexuality and reproduction [1,8,9]; both access and freedom are highly influenced by social factors such as socioeconomic status and gender relations [10].

Though there is considerable evidence regarding the effect of individual risk factors on unintended pregnancies [11,12], the role of the social environment in which those pregnancies occur has hardly been explored [13]. A recent study, based on the 1998 and 2003 Ghana Demographic and Health Surveys, showed a geographical heterogeneity in the risk of mistimed and unwanted pregnancy after controlling for relevant predictors. The study observed a uniquely high concentration of mistimed pregnancies in some rural communities relative to others, and a marked variation in the risk attached to unwanted pregnancy between urban communities [14].

In Ecuador, recent research suggests that 36.3% of all births are unintended (18.7% unwanted and 17.6% mistimed). Differences between geographical areas are prominent, with the Amazon Basin displaying the highest percentage of unintended pregnancies (43.3%) [15]. Studies forming a part of the 1994 Ecuador Demographic and Maternal and Child Health Survey reported a connection between unwanted pregnancy -- but not mistimed pregnancy - and lower levels of prenatal care (delayed care and a number of visits below that recommended) [16] and increased risk of low birth weight [17]. The likelihood of a pregnancy being labelled as unwanted increases with maternal age (48.6% among women age 40-49), and the number of already existing children (50.8% among women with 7 or more children). Unwanted pregnancy is more common among illiterate women (28.8%), and women living in the poorest households (23.4%); this reflects the connection between effective reproductive planning and social determinants of reproductive health [15]. These studies focused on individual risk factors and were based on large national surveys where local samples, particularly from remote rural areas, were small. A greater understanding of local contextual factors has the potential to provide better information to decision-makers involved in sexual and reproductive health community-level programs.

The purpose of this study was to estimate the effect of community factors and individual characteristics on self-reported unintended pregnancies in the Amazon Basin of Ecuador. To accomplish this, a multilevel logistic regression analysis to account for individual-level and community-level variables was used.

## Methods

### Study site

Orellana, with 103,032 inhabitants, is a province located in the Amazon Basin of the country. Its population is primarily rural, with only 30% living in urban areas. The province is divided into four counties. For this study, two settlements were considered urban areas: the capital, Coca, with approximately 20,000 inhabitants; and Sachas, with 7,000 inhabitants. In the rural areas, people usually live in small communities, ranging from 300 to 500 people. Houses in these communities are typically separated by some distance, and each community has a small centre where the school is based. Thirty percent of Orellana's population is indigenous (mainly Kichwa) and is concentrated in rural indigenous communities where land ownership is communal. In urban and rural neighbourhoods, the proportion of indigenous people is very low. Almost 50% of the population is under the age of 15 years.

Responsibility for public health in Orellana lies with the Ministry of Health's provincial department. There is a small, 20-bed hospital in Coca, three health centres based in the largest towns, and small health facilities in rural areas. Around half of the rural communities also have a health post, managed by community health workers (volunteers chosen from within the community and who have had preventive and curative medical training). Emergency transport is difficult because infrastructure is poor. When patients cannot be treated at the provincial hospital, they are referred to the capital Quito, 350 km away.

A cross-sectional survey, carried out in Orellana in 2006, evidenced that the prevalence of unwanted pregnancy in Orellana appeared to be much higher than the national average, and remarkable differences existed between indigenous women - who accounted for 43.6% of unwanted pregnancies - and the rest (30.6% for non-indigenous women living in rural areas and 29% for those living in urban areas) [18]. Those results motivated the present analysis, which was designed to explore in greater detail the connection between unwanted pregnancy, ethnical differences, and their potential risk factors.

### Study population

The study population consisted of women between 15 and 44 years living in the province of Orellana. The women were selected from an ongoing community-based cross-sectional survey that was conducted in the province between May and December 2006. The selection of women followed a two-stage cluster sampling procedure. A list of communities was developed using data from local government, including approximate information on the number of inhabitants that were women. Clusters with a similar number of inhabitants were created by gathering together small communities, or by splitting big communities into several clusters of the same size. After constructing the clusters, a number of them were randomly selected until reaching the sample size that was needed. The selected communities were visited and all the houses within the community were included in the survey. In rural areas, a community was defined as a group of households that followed a political boundary. In urban areas, a community was a neighbourhood.

Women without children, pregnant women (n = 125), and women breastfeeding at the time of the interview (n = 73) were excluded from the analysis. The last two groups were not included to avoid respondent bias - the rational for this was that the feelings about pregnancy of women who were pregnant or breastfeeding (and thus in a close relationship with the baby), might be different from those of women whose last delivery occurred at least one year before. Because of the illegality of induced abortion in Ecuador, only women who had delivered a child were included. The final sample consisted of a total of 1,002 women (90.5% of the sample of 1107 women aged 15-44, which fulfilled the inclusion criteria) from 34 communities.

### Data collection

Structured interviews were conducted in Spanish by female field workers using a two-part questionnaire. Interviews were held in the houses of the respondents. When the respondent was from the Kichwa ethnic group, the interview was conducted in their native language, in order to ensure that the questions were fully understood. The first part of the questionnaire recorded household socioeconomic and demographic information. The second part was administered only to women aged 15--44, obtained information on fertility, all the pregnancies each woman had experienced, as well as details about contraceptive use and pregnancy intention.

### Variables

The dependent variable was the reported unintended pregnancy (both unwanted and mistimed pregnancies were included in this category). Women were asked regarding their last pregnancy: "When you discovered that you were pregnant, what did you think?" Four possible answers to this question were provided:

1) I wanted the pregnancy at that very time.

2) I would have wanted the pregnancy to happen later.

3) I did not want the pregnancy at all.

4) I don't know or remember.

Unintended pregnancy (answers 2 and 3) was classified as a dichotomous variable (yes/no).

Several variables that have been associated with unintended pregnancy in the literature were included in the study as independent variables; they were grouped into individual-level and community-level factors.

Individual-level variables included were age (grouped as 15-19, 20-29, 30-39, 40-45), civil status (single, married/union, other), education (non-primary, primary, more than primary), number of living children (categorized as 0-1, 2-3, >3), ethnic group (indigenous or non-indigenous), current use of contraceptives (yes/no), and use of modern contraceptives (yes/no). Reasons for not using contraception, including, among other possibilities, current pregnancy or recent delivery, were also ascertained. 'Modern contraceptives' included: hormonal methods (oral or injection), intrauterine devices, sterilization, and condoms. Herbal remedies and the rhythm method were excluded from this category. A proxy for socioeconomic status was estimated using principal component analysis of 20 variables, including access to water, sanitation, and household characteristics (materials used to build floor, roof and walls). The first principal component was divided into quintiles, so that each household was classified as: most poor, very poor, poor, less poor, or least poor in terms of socioeconomic status [19].

Four community-level variables were included: geographical location (urban, rural), type of community (neighbourhood urban, neighbourhood rural, non-indigenous rural community, and indigenous rural community), community education (defined as the percentage of women with more than primary education, coded in two classes with the median value as the cut-off), and community wealth (defined as the median value of the individual socioeconomic index for all participants in a community, and classified in quartiles). The last two variables were derived from data on the whole population of the community.

### Statistical analysis

The descriptive statistics show the distribution of respondents by the key variables. Values were expressed as an absolute number (percentages) and as a mean (SD) for categorical and continuous variables, respectively. We conceptualized the analysis in a multilevel structure, comprising individuals (at level 1) nested within communities (at level 2). We fitted the data using multilevel logistic regression, adjusting for both individual-level and community-level factors as fixed effects and allowing for heterogeneity between communities. The two-level model was specified with a binary response (whether or not it was an unintended pregnancy) for a woman living in a community.

The analysis was done in four steps: In Model 1 (empty model), no explanatory variable was included. This model represented the total variance in unintended pregnancies between the communities. In Model 2, only individual-level factors were included to test the extent to which community-level differences were explained by individual factors of the communities. Model 3 is about the effects of community factors on unintended pregnancies, and Model 4 expands on the previous model by adding the individual-level variables. The results of fixed effects (measures of association) were shown as odds ratios (ORs) with 95% confidence intervals (CIs). Non-significant variables (p > 0.10) were excluded from the final model. The results of random effects (measures of variation) were presented as the variance partition coefficient (VPC) and percentage change in variance (PCV). In logistic regression models, the VPC is calculated as V_A_/(V_A_+3.29) where V_A _is the community-level variance. The median odds ratio (MOR) has also been postulated as an appropriate way of translating the area-level variance into the widely used odds ratio. The MOR is defined as the median value of the odds ratio between the area at highest risk and the area at lowest risk when randomly picking out two persons from those areas. Large MOR indicates differences between areas in the probability of the outcome, pointing to the relevance of the area level for understanding variation of the individual probability of the outcome. MOR was computed with the formula MOR = exp(0.95√V_A_) [20]. Statistical analyses were performed using Stata 10.0 with the command xtmelogit (Stata Corp. Inc., TX, USA).

### Ethics

Although there was no local ethics committee in the area, several considerations were taken onboard to ensure compliance with ethical principles. The provincial authorities approved the development of this study and, before the survey was conducted, community leaders were informed and asked for their cooperation. Informed consent was obtained from all participants in the survey.

## Results

### Characteristics of the populations

A total of 1,002 women nested within 34 communities participated in the study, with the number of women per community ranging from 14 to 94. Table 1 shows the distribution of individual-level and community-level characteristics. Most of the women belonged to the age group 20-39, and had finished primary education. More than two-thirds of the participants were married or in a union (84.04%) and belonged to the non-indigenous group (65.47%). Self-reported use of contraceptives was high, with nearly half (47.7%) using modern contraceptives. Most of the population was rural (60.48%), approximately half of which was comprised of indigenous people.

**Table 1:** Sociodemographic characteristics of participants and percentage of women reporting unintended pregnancy by individual-level and community-level variables

*Individual variables*	Number of women (%)		Proportion of unintended pregnancy
**Age groups**	1,002		

15-19	113	(11.3)	55.7

20-29	411	(41.0)	60.8

30-39	352	(35.1)	65.3

=>40	126	(12.6)	68.2

**Level of education**	979		

No school	128	(13.1)	72.7

Primary	432	(44.1)	68.7

More than primary	419	(42.8)	54.2

**Marital status**	990		

Married/union	832	(84.0)	62.0

Single	116	(11.7)	67.2

Other	42	(4.2)	61.9

**Occupation**			

Housekeeper	704	(71.3)	61.9

Student	41	(4.1)	70.7

Farmer	74	(7.5)	74.3

Other	169	(17.1)	59.8

**Ethnic group**	1002		

Non-indigenous	656	(65.5)	57.0

Indigenous	346	(34.5)	73.7

**No. of living children**	1,002		

1-2	431	(43.0)	49.6

3-4	301	(30.0)	66.4

=>4	270	(26.9)	79.6

**Wealth index**	1,002		

1 (Low)	250	(24.9)	68.0

2	264	(26.3)	67.8

3	224	(22.4)	61.2

4 (High)	264	(26.3)	54.2

**Use of contraception**	997		

1 = Yes	746	(74.8)	63.0

2 = No	251	(25.2)	61.7

**Use of modern methods**	1,000		

1 = Yes	477	(47.7)	61.0

2 = No	523	(52.3)	64.2

			

*Community variables*			

**Place of residence**	1,002		

Rural	606	(60.5)	66.3

Urban	396	(39.5)	57.3

**Type of community**	1,002		

Neighbourhood urban	395	(39.4)	57.2

Neighbourhood rural	151	(15.1)	63.6

Non-indigenous community	167	(16.7)	56.9

Indigenous community	289	(28.8)	73.4

**Education**			

Low	508	(50.7)	66.3

High	494	(49.31)	59.1

**Socioeconomic index**			

1 (Low)	278	(27.7)	71.6

2	260	(25.9)	65.8

3	215	(21.5)	59.1

4 (High)	249	(24.8)	53.0

The overall prevalence of unintended pregnancies was 62.7%. Around one-third of women (35.3%) expressed that they had not wanted to become pregnant and another third (27.4%) that they would have wanted to delay the pregnancy. Two-thirds (73.7%) of indigenous women reported unintended pregnancies (44.8% unwanted, and 28.9% mistimed). Table 1 also presents the prevalence of unintended pregnancy according to the different individual and community variables. To be indigenous, to be a farmer, not to have gone to school, to have more than four children, and to belong to an indigenous community -- these variables characterised the groups with the highest proportions of unintended pregnancy.

### Determinants of unintended pregnancy

The results of the Random Intercept Only model are shown in Table 2 (Empty Model 1). There was a significant variation in the log odds of unintended pregnancy across the communities (V_A _= 0.115; p = 0.001). According to the VPC, 3.37% of the total individual differences between unintended pregnancies were at the community-level. The MOR (the residual heterogeneity between areas) was 1.38, which indicated small differences between communities in the probability of unintended pregnancy occurring.

**Table 2:** Multilevel logistic regression of the association between individual and community characteristics and unintended pregnancy in Orellana^a^

	**Model 1**^**b**^	**Model 2**^**c**^	**Model 3**^**d**^	**Model 4**^**e**^
***Individual variables***		**OR (95% CI)**	**OR (95% CI)**	**OR (95% CI)**

**Age groups**				

=>40		ref		ref

30-39		1.11 (0.68-1.80)		1.13 (0.70-1.84)

20-29		**1.75 (1.04-2.93)**		**1.78 (1.06-3.00)**

15-19		**2.31 (1.21-4.40)**		**2.34 (1.22-4.47)**

**Level of education**				

More than primary		ref		ref

Primary		**1.48 (1.09-2.0)**		**1.53 (1.12-2.10)**

No school		**1.82 (1.14-2.89)**		**1.89 (1.18-3.03)**

**Marital status**				

Married/union		ref		Ref

Single		**1.98 (1.27-3.09)**		**2.00 (1.28-3.11)**

Other		1.01(0.51-1.98)		0.98 (0.50-1.94)

**Ethnic group**				

Non-indigenous		ref		ref

Indigenous		**1.65 (1.21-2.24)**		**2.01 (1.10-3.65)**

**No. of living children**				

1-2		ref		ref

3-4		**2.55 (1.77-3.67)**		**2.56 (1.77-3.70)**

>4		**5.29 (3.28-8.52)**		**5.46 (3.38-8.48)**

*Community variables*				

**Ethnic community**				

Neighbourhood urban			ref	ref

Neighbourhood rural			1.31 (0.84-2.04)	0.82 (0.53-1.27)

Non-indigenous rural			0.95 (0.63-1.42)	0.84 (0.55-1.27)

Indigenous rural			**2.02 (1.40-2.92)**	0.72 (0.37-1.40)

*Random effects*				

Community random variance (SE)^f^	0.11 (0.06)	0.00 (0.00)	0.003 (0.034)	0.00 (0.00)

VPC (%)^f^	3.37	0.0	0.09	0.0

PCV (%)^f^	reference	100	97.39	100

MOR^f^	1.38	1.00	1.05	1.00

^a ^Variables that showed statistically significant association are in bold.

^b ^Model 1 presents a null model with no fixed-effect predictors and a random variance component for communities.

^c ^Model 2 presents individual predictor variables in the fixed part and a random variance at community level.

^d ^Model 3 presents community predictor variables in the fixed part and a random variance at community level.

^e ^Model 4 presents both individual and community predictor variables in the fixed part and a random variance at community level.

^f ^SE= Standard error; VPC= Variance partition coefficient; PCV=Percentage change in variance; MOR= Median odds ratio.

To assess if differences between communities were attributable to the individual composition of the communities or caused by a true contextual effect, an adjustment for individual factors was made. The results of fitting the model including individual-level variables appear in Table 2 (Model 2). The two youngest groups of women (15-19, 20-29), low education, and being single, were all significant risk factors for unintended pregnancies. To be indigenous, and the number of existing children were also statistically significant. No relationship was found between socioeconomic factors and the use of contraception.

The inter-community variance decreased from 0.11 to practically zero, indicating that all the variation in unintended pregnancy between communities was explained by differences in their individual factors. The clustering of women within communities with respect to having unintended pregnancy vanished (VPC = 0.00, MOR = 1.00), and this model explained practically all such differences between communities.

The variable *type of community *was the only contextual variable associated with unintended pregnancy (model 3); this association, however, disappeared when individual-level variables were included in the model (model 4). Place of residence (urban/rural), and the education and socioeconomic indexes were excluded from the model, since they were not significant.

## Discussion

This study examined risk factors for unintended pregnancy among women in the Amazon Basin of Ecuador using a multilevel analytical framework. The findings of this study show that a high proportion of women, particularly indigenous, would have preferred to avoid their last pregnancy. All the variation between communities was explained by individual-level factors.

Total fertility rates for Ecuador have been steadily declining, and this has been regarded as a direct indicator of the success of interventions aimed at improving access to contraception across the country. Total fertility rates are useful in evaluating the effect of population policies and demographic goals, but less informative when assessing the extent to which individual women are exercising their right to decide when they get pregnant; for this last purpose, *pregnancy intention *seems to be a more accurate indicator. While total fertility rates have been declining from 3.8 in the period 1984-1989 to 3.3 in 2004, the prevalence of unintended pregnancy has actually increased: in the 1989 ENDEMAIN (a national demographic, maternal and infant health survey that is carried out every four years), 13% of women in unions reported their pregnancy as unintended compared to 36.3% in the most recent 2004 ENDEMAIN [11,15]. National statistics also fail to point out which women experience the highest proportions of unintended pregnancies and, consequently, programs fail to reach them with suitable interventions. While national statistics report that 43.3% of pregnancies in the Amazon region are unintended, our findings present an even more dismal situation: 62.7% of pregnancies are unintended (35.3% unwanted and 27.4% mistimed).

This study also sheds some light on the factors that increase the woman's risk of experiencing an unintended pregnancy, evidencing that these are not random events. Indigenous women, women who were young, single, low educated, and already with more than two children (and especially if they had more than four) had a significantly higher likelihood of having an unintended pregnancy. The *type of community *variable was the only contextual factor associated with unintended pregnancies, but the association disappeared when individual-level variables were included. Given that the variation between areas was extremely low, it could hardly explain anything. Most of these risk factors have also been identified in previous studies from Ecuador [11] and elsewhere [21].

It is interesting to note that while the youngest women (15-19 years old) reported the lowest proportion of unintended pregnancy in the univariate analysis, when adjusting for the number of children, this age group showed the highest risk of experiencing an unintended pregnancy. When the effect of the number of children was controlled, more young women (already with a child) preferred not to have had the last child compared to the older ones. This finding is highly relevant because it challenges the assumption made by many programs for adolescent pregnancy prevention, which consider this age group as low-risk for unintended pregnancy [10].

The effect of marital status is not surprising, and in fact the stigma associated with single motherhood has been evidenced in past qualitative research [10,22]. From a gender perspective, the relationship between marital status and pregnancy intention, and also the problematization of pregnancy based not on a pathological condition but on a social category, deserve further research. It could also be interesting to explore more in depth the effect of gender inequality and machismo on women's decision making regarding sexuality and reproduction. The fact that both mistimed and unwanted pregnancies were common among those women might be a sign of a gender inequality that curtails women's sexual and reproductive autonomy [10].

The increased risk of unintended pregnancy among women who already had more than two children could be a signal of the disconnection between maternal and child care, and family planning services. Women that have children are more likely to attend health services, if not for delivery (access to skilled delivery is quite low in this area, especially for indigenous women) then for prenatal checkups or infant care [18]. Thus, in Orellana, either these services did not take this opportunity to meet these women's need for family planning, counselling and provision, or they did it in an ineffective way. It could also be the consequence of the vicious circle of unintended pregnancy, resulting in a diminished likelihood of accessing health services for prenatal and infant checkups [16], and consequently a diminished opportunity to receive counselling on how to prevent subsequent unintended pregnancies.

The effect of ethnicity deserves special attention. It was the only determinant that had influence at both the individual and the community levels (though not significantly in the latter case). In this study, both variables were measuring practically the same, since almost all indigenous women live in indigenous communities. From a rights perspective, this is most likely a sign of indigenous women suffering from unequal access to the means that could enable them to realize their reproductive rights - dependent not only on the network of services they are entitled to, but also on the social determinants affecting their reproductive health [3,8,9,23]. This unequal access could be due not only to the unequal distribution of resources such as health centres and medical staff, but also to the fact that policies and programs are not designed from an indigenous perspective and thus might fail to ensure cultural accessibility [18,24-26]. The differentiated effect of gender relations on indigenous women could also be a reason behind their increased risk of unintended pregnancy. Our findings also challenge the assumption that indigenous women want to have more children because of their cultural traditions - both issues definitely require further research.

It is also interesting to bring up two of the determinants that did not show an effect on the risk of unintended pregnancy: poverty, and use of modern contraceptive methods. Poverty has been linked with unintended pregnancy in previous research, but was not found to be significant in this study - this could be a reflection of the generally very low socioeconomic status of the majority of households in Orellana province, independently of them being in rural or urban areas and in indigenous or non-indigenous communities.

The fact that the reported actual use of modern contraceptives did not reduce the risk of experiencing unintended pregnancy, seems to contradict the well-established fact that increasing use of modern contraceptives is an effective intervention for decreasing unintended pregnancies [27-29]. In this case, it could be a reflection of a somehow incorrect use of the particular contraception (the most prevalent modern methods were hormonal methods, and they could fail if not administered or taken at specific times); also, since *pregnancy intention *referred to a past event, while *use of modern methods *referred to the present status when the interview was conducted, it could reflect a negative memory of a previous unintended pregnancy on the women deciding to initiate family planning. It could also be due to the effect of sexual violence and intimate partner violence, as unwanted and unprotected sexual intercourse can often result in very much unwanted pregnancies that cannot be legally aborted (in Ecuador, abortion is legally permitted only to save the woman's life or when the pregnancy is as a result of the rape of a mentally disabled woman). In fact, the association between intimate partner violence and unintended pregnancy has been already found by other authors [30], and previous studies in Orellana [31].

There are several limitations to be considered when interpreting these results, such as the cross-sectional design of the study, which limits its ability to draw causal inferences. In addition, information regarding both the outcome and the different independent variables was self-reported and therefore subject to reporting bias, particularly in the report of unintended pregnancies. A potential bias could also come from women's reluctance to classify their offspring as unwanted, for example, due to social stigmatisation. Bias could also have occurred because the woman could have changed her perception of the pregnancy over time and, as a consequence, pregnancies that could have initially been labelled as *unintended *could be perceived retrospectively as *wanted*. However, the estimates of the determinants would only be affected if such misclassification was selective by the characteristics under investigation. Another potential bias might be related to the exclusion criteria - since we only included women with children, women who had undergone an abortion were excluded. The decision to exclude those women was taken because of the illegality of abortion in Ecuador, and because of the stigma associated with it, which would have complicated the collection of information. The fact that we also excluded women who were pregnant and women who were breastfeeding at the time, could have also affected the results. Community was chosen as the geographical unit of analysis. These units represent the smaller area level and constitute stable socio-political and geographic entities, within which social and public health policies are formulated. It may be that bigger geographical areas are more suitable for the analysis of contextual influence on unintended pregnancy.

## Conclusions

In Orellana, women in general reported a large number of unintended pregnancies (62.7%) and this necessitates a stronger promotion of women's reproductive rights to decide regarding when (if ever) and with whom, they want to get pregnant and raise children.

This is especially relevant in places such as Orellana, where unintended pregnancies might not only negatively affect the health of the new born, but might even pose a risk to the mother's life, because the criminalization of abortion increases the risk of resorting to unsafe procedures, especially for poor women [29,32]. Since it seemed that family planning strategies were not working, qualitative research might shed more light on how health services can make contraception more accessible and relevant to women living in this area. Though this study has shown a lack of variability in unintended pregnancy at the community level (pointing towards individual rather than community-based interventions), for all women to be able to realize this reproductive right, approaches need to be diverse; the needs of young, single women - probably seeking to delay pregnancy - will likely differ markedly from the needs of adult women with many children, who may want to avoid becoming pregnant ever again. In order to be successful in reaching indigenous women, interventions should also consider strengthening cultural accessibility.

## Competing interests

The authors declare that they have no competing interests.

## Authors' contributions

IG supervised the data collection, participated in the analysis and in the writing of the manuscript. MSS worked on the analysis of the data and in the writing of the manuscript. Both authors read and approved the final manuscript.
